# Risk factors for acute kidney injury in critically ill patients receiving high intravenous doses of colistin methanesulfonate and/or other nephrotoxic antibiotics: a retrospective cohort study

**DOI:** 10.1186/cc12853

**Published:** 2013-08-14

**Authors:** Monica Rocco, Luca Montini, Elisa Alessandri, Mario Venditti, Amalia Laderchi, Pascale De Gennaro, Giammarco Raponi, Michela Vitale, Paolo Pietropaoli, Massimo Antonelli

**Affiliations:** 1 Anesthesiology and Intensive Care, Sapienza University of Rome, Viale del Policlinico 155, 00161 Rome, Italy; 2 Anesthesiology and Intensive Care, Catholic University of the Sacred Heart, Largo Agostino Gemelli 8, 00168 Rome, Italy; 3 Department of Public Health Sciences and Infectious Diseases, Sapienza University of Rome, Viale del Policlinico 155, 00161 Rome, Italy

## Abstract

**Introduction:**

Use of colistin methanesulfonate (CMS) was abandoned in the 1970s because of excessive nephrotoxicity, but it has been reintroduced as a last-resort treatment for extensively drug-resistant infections caused by gram-negative bacteria (*Acinetobacter baumannii, Pseudomonas aeruginosa, Klebsiella pneumonia*). We conducted a retrospective cohort study to evaluate risk factors for new-onset acute kidney injury (AKI) in critically ill patients receiving high intravenous doses of colistin methanesulfonate and/or other nephrotoxic antibiotics.

**Methods:**

The cohort consisted of 279 adults admitted to two general ICUs in teaching hospitals between 1 April 2009 and 30 June 2011 with 1) no evidence on admission of acute or chronic kidney disease; and 2) treatment for more than seven days with CMS and/or other nephrotoxic antimicrobials (NAs, that is, aminoglycosides, glycopeptides). Logistic regression analysis was used to identify risk factors associated with this outcome.

**Results:**

The 279 cases that met the inclusion criteria included 147 patients treated with CMS, alone (n = 90) or with NAs (n = 57), and 132 treated with NAs alone. The 111 (40%) who developed AKI were significantly older and had significantly higher Simplified Acute Physiology Score II (SAPS II) scores than those who did not develop AKI, but rates of hypertension, diabetes mellitus and congestive heart failure were similar in the two groups. The final logistic regression model showed that in the 147 patients who received CMS alone or with NAs, onset of AKI during the ICU stay was associated with septic shock and with SAPS II scores ≥43. Similar results were obtained in the 222 patients treated with CMS alone or NAs alone.

**Conclusions:**

In severely ill ICU patients without pre-existing renal disease who receive CMS high-dose for more than seven days, CMS therapy does not appear to be a risk factor for this outcome. Instead, the development of AKI was strongly correlated with the presence of septic shock and with the severity of the patients as reflected by the SAPS II score.

## Introduction

Throughout the world, *Acinetobacter baumannii, Pseudomonas aeruginosa *and *Klebsiella pneumonia *have emerged as major causes of nosocomial infections [1], particularly in patients who are critically ill and/or immunocompromised. Concern has been raised by reports of a stepwise trend towards extensive drug-resistance in these organisms [1]. Infections caused by extensively drug-resistant (XDR) bacterial strains are associated with high mortality rates, especially in intensive care units (ICUs), where outbreaks are extremely difficult to control. The limited therapeutic options in these cases often lead clinicians to resort to salvage therapy with colistin methanesulfonate (CMS). This older polymyxin antibiotic, which is converted *in vivo *to colistin [2], was widely abandoned in the 1970s because of its unfavorable pharmacokinetic properties and frequent adverse effects, particularly nephrotoxicity.

The "modern polymyxin era" [3], which began in the late 1990s, is characterized by a variety of dosing schedules, but to date there is still a dearth of information on the clinical pharmacokinetics of CMS and colistin in critically ill patients [4]. Higher doses appear to be beneficial in these cases [5], but it is unclear whether the improved efficacy comes at a cost of increased toxicity. The aim of this retrospective cohort study was to evaluate the potential risk factors for acute kidney injury (AKI), as defined by the RIFLE (Risk of renal dysfunction, Injury to the kidney, Failure of kidney function, Loss of kidney function, End-stage kidney disease) classification system [6], in severely ill ICU patients without pre-existing renal disease who received high-dose intravenous CMS therapy for more than seven days.

## Materials and methods

This study was conducted in two large tertiary-care teaching hospitals in Rome, Italy (Policlinico Umberto I and the Policlinico Gemelli), and it involved retrospective analysis of prospectively collected data. Cases were identified through searches of the ICU patient databases, and data were collected from the patients' electronic medical records.

**Figure 1:**
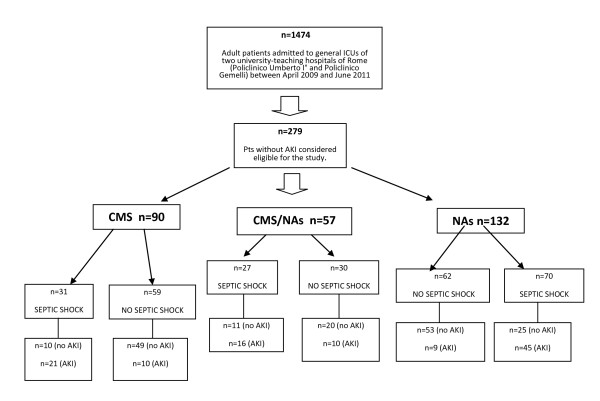
**Study design**. AKI, acute kidney injury (defined according to RIFLE criteria); CMS, colistin methanesulfonate sodium; NAs, nephrotoxic antibiotics (aminoglycosides, glycopeptides); Pts, patients

The study cohort consisted of adults (≥18 years) consecutively admitted to the general ICUs of the participating facilities between April 2009 and June 2011 (Figure 1). Inclusion criteria were: 1) no evidence on ICU admission - as well as at protocol admission - of chronic renal failure and normal estimated glomerular filtration rate (GFR) relative to serum creatinine (SCr) based on age, race and sex formula assuming a glomerular filtration rate of 75 mL/min/1.73 m^2^, as recommended by the Acute Dialysis Quality Initiative (ADQI) Working Group [6]. Most ICU patients, in fact, have not a prior measure of renal function and a simplified modification of diet in renal disease (MDRD) formula provides a simple and precise estimation of baseline GFR and SCr 2) onset >48 h after ICU admission of an XDR bacterial infection treated for seven or more days with intravenous (iv) CMS and/or other nephrotoxic antimicrobial agents (NAs, that is, aminoglycosides and glycopeptides).

Extensively drug-resistant (XDR) was defined as non-susceptibility to at least one agent in all but two or fewer antimicrobial categories (that is, bacterial isolates remain susceptible to only one or two categories) [7]. Patients were excluded if the antibiotic therapy described above had been started prior to ICU admission.

The primary end point of the study was to evaluate the potential risk factors for acute kidney injury (AKI) in severely ill ICU patients without pre-existing renal disease who received high-dose intravenous CMS therapy with or without other nephrotoxic antimicrobials.

For this purpose, patients were classified daily using the RIFLE criteria and AKI was defined using the serum creatinine compared to the baseline value of the SCr previously obtained from the MDRD equation.

**Figure 2:**
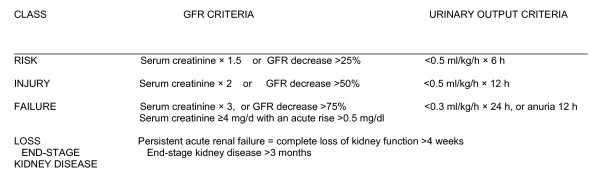
**RIFLE classification**. Patients are classified on serum creatinine or urinary output, or both, the worst parameters are used. Glomerular filtration rate (GFR) criteria are calculated as an increase of serum creatinine above the baseline serum creatinine level. When the baseline serum creatinine is unknown and there is no past history of chronic kidney disease, serum creatinine is calculated using the Modification of Diet in Renal Disease formula for assessment of kidney function assuming a GFR of 75 ml/min/1.73 m^2^. RIFLE, Risk Injury-Failure-Loss-End-stage kidney disease

A patient was considered to have AKI when he had an increase in SCr of at least 50% from baseline (defined as Risk) or if he doubled the SCr level from the baseline (defined as Injury) or had a three times increase in SCr (defined as Failure) [6,8] (Figure 2).

For each patient included in the cohort, electronic hospital charts were reviewed, and the following collected data were recorded: demographic variables; Simplified Acute Physiology Score II (SAPS II); presence on admission of hypertension, diabetes mellitus, and/or congestive heart failure; reason for ICU admission; length of ICU stay; type and cause of infection; nephrotoxic drugs and iv iodate contrast used, immunocompromised status; albumin serum level, bilirubin serum level and, for CMS, duration of therapy and cumulative doses; presence of septic shock caused by the XDR infection; use of continuous renal replacement therapy (CRRT) during the ICU stay and ICU mortality.

Septic shock was diagnosed as a state of acute circulatory failure characterized by persistent arterial hypotension despite adequate fluid resuscitation or by tissue hypoperfusion in the presence of proven or suspected infection [9]. Bloodstream infection (BSI) was defined as at least one positive blood culture for a potential bacterium together with clinical features compatible with systemic inflammatory response syndrome; the clinical suspicion of pneumonia was based on either clinical criteria (new or progressive radiologic pulmonary infiltrate together with at least two of the following: temperature >38°C or <36°C, leukocytosis >12,000/mL or leucopoenia <4,000/mL, or purulent respiratory secretions) or a simplified Clinical Pulmonary Infectious Score greater than or equal to six points. The microbiologic evaluation included the collection of at least one lower respiratory airway sample by tracheobronchial aspirates, bronchoscopic or blind bronchoalveolar lavage, within the first 24 hours of the onset of symptoms. Microbiologic confirmation of pneumonia was defined by the presence of at least one potentially pathogenic microorganism in respiratory samples above predefined thresholds bronchoalveolar lavage >10^4^, and sputum or tracheobronchial aspirates >10^5 ^colony-forming units/ml, respectively [10,11].

This study was approved by our institutional review board that waived the need for informed consent, due to the retrospective design of this study.

### Statistical analysis

MedCalc software, version 12.1.0 (MedCalc^® ^Software v 12.2.1, MariaKerke, Belgium) was used for all statistical analyses. Differences between groups were assessed with the Mann-Whitney test and results given as medians and interquartile ranges (IQR). The Kolmogorov-Smirnov test was used to assess variable distribution. Categorical variables, presented as proportions, were analyzed with the chi-square test or Fisher's exact test, as appropriate. *P-*values of <0.05 were regarded as significant. Potential risk factors for AKI were identified by means of univariate analysis with calculation of crude odds ratios (ORs). Those that emerged from this analysis with a *P-*value of <0.2 were candidates for inclusion in the multivariate model. The variables included in the final predictive model were selected with a stepwise procedure, and the accuracy of the model was assessed in terms of the area under the receiver operating characteristic (ROC) curve.

## Results

Between April 2009 and June 2011, 1,474 adult patients were consecutively admitted to the two participating ICUs, and 279 (19%) of these met the criteria for inclusion in the study (Figure 1). Their characteristics are reported in Tables 1 and 2. The NAs patients were older, had a longer ICU stay and had a higher percentage immunocompromised than the other groups; the number of NAs patients treated with nonsteroidal anti-inflammatory drugs (NSAIDs) were statistically significant in respect to the CMS group (Table 1).

**Table 1:** ACEI, angiotensin converter enzyme inhibitor; AKI, acute kidney injury as defined per RIFLE criteria; BMI, body mass index; BSI, bloodstream infection; CMS, colistin methanesulfonate sodium; CRRT, continuous renal replacement therapy; CVC, central venous catheter; NSAID, nonsteroidal anti-inflammatory drug; Pts, patients; SAPS II, simplified acute physiology score two (calculated 24 h after ICU admission); VAP, ventilator-associated pneumonia.

VARIABLES	CMS (n = 90)	CMS + NAs (n = 57)	NAs (n = 132)
Age (years)	57 (40 to 69)	54 (39 to 66)	67 (48 to 76) ^*^
Female, n (%)	32 (35)	20 (35)	40 (30)
SAPS II	41 (32 to 54)	44 (30 to 54)	44 (35 to 55)
BMI, Kg/m^2 ^	25 (24 to 25)	24 (23.7 to 25)	24 (23 to 27)
ICU length of stay, (days)	28 (17 to 38)	33 (19 to 50)	15 (8 to 31) *
Albumin serum level <2 g/dL, n (%)	14 (15)	9 (16)	18 (14)
Total bilirubin serum level >5 mg/dL n (%)	7 (8)	4 (7)	11 (8)
NSAID n (%)	17 (19)	10 (18)	43 (33) ****
ACEI n (%)	15 (17)	3 (5)	16 (12)
i.v. iodate contrast n (%)	35 (39)	14 (25)	45 (34)
Immunocompromised pts n (%)	13 (14)	8 (14) ***	38 (29) ^* ^*
**Reason for ICU admission**			
Sepsis, n (%)	41 (46)	20 (35)	56 (43)
Neurological injury, n (%)	4 (4)	8 (14)	12 (9)
Traumatic injury, n (%)	31 (34)	21 (37)	31 (23)
Cardiovascular injury, n (%)	14 (16)	8 (14)	33 (25)
**Comorbidity **			
Hypertension, n (%)	14 (16)	12 (21)	28 (21)
Diabetes mellitus, n (%)	2 (2)	2 (3.5)	9 (7)
Congestive heart failure, n (%)	3 (3)	2 (3.5)	10 (7.5)
Two or more comorbidities, n (%)	5 (5)	4 (7)	16 (12)
**Site of infection**			
VAP, n (%)	69 (77)	40 (70)	91 (69)
CVC related-BSI, n (%)	18 (20)	13 (23)	31 (23)
Other, n (%)	3 ^a ^(3)	4^b ^(7)	10 ^c ^(7)
**Complications occurring during ICU stay**			
CRRT, n (%)	13 (14)	14 (24)	29 (22)
AKI, n (%)	31 (34)	26 (45)	54 (41)
Septic shock, n (%)	31 (34)	27 (47)	70 (53)
ICU mortality, n (%)	31 (34)	21 (37)	64 (49)

Other nephrotoxic antimicrobial included aminoglycosides and glycopeptides.

**^(a) ^**wound infection n = 2, abdominal abscess n = 1. ^(b) ^wound infection n = 1, abdominal abscess n = 1, urinary tract infection n = 2. ^(c) ^urinary infection n = 4, abdominal abscess n = 3, wound infection n = 1, meningitis n = 2. Values are given as the median (interquartile range).

^* ^*P *<0.01 NAs *vs *CMS and CMS/NAs; ** *P *= 0.01 CMS vs NAs; ^*** ^*P *= 0.04 CMS/NAs vs NAs *P *= 0.03 ****

**Table 2:** ACEI, angiotensin converter enzyme inhibitor; AKI, acute kidney injury; BSI, bloodstream infection; CMS, colistin methanesulfate; CVC, central venous catheter; NAs, other nephrotoxic antibiotics (aminoglycosides, glycopeptides); NSAID, nonsteroidal anti-inflammatory drug; SAPS II, Simplified Acute Physiology Score Two (calculated 24 h after ICU admission); VAP, ventilator-associated pneumonia.

Variables	Total cohort (n = 79)	No AKI (n = 168)	AKI (n = 111)	*P*-value ^a^
Age, years ^b^	61 (43 to 74)	58 (39 to 71)	66 (51 to 77)	<0.01 *
Female, n (%)	92 (33)	62 (37)	30 (27)	0.09 **
SAPS II score ^b^	44 (32 to 54)	38 (29 to 49)	50 (41 to 56)	<0.01 *
Septic shock at infection onset - n (%)	128 (46)	46 (27)	82 (74)	<0.01 **
Albumin serum levels <2 g/dL n (%)	38 (14)	15 (9)	23 (21)	<0.01 **
Total bilirubin serum levels >5 mg/dL n(%)	19 (7)	8 (5)	11 (10)	0.15 **
NSAID n (%)	70 (25)	48 (28)	22 (20)	0.13 **
ACEI n (%)	34 (12)	16 (10)	18 (16)	0.13 **
Immunocompromised pts n (%)	59 (21)	34 (20)	25 (23)	0.75 **
i.v. iodate contrast n (%)	94 (34)	53 (32)	41 (37)	0.42 **
**Reason for ICU admission **				
Sepsis, n (%)	117 (42)	60 (36)	57 (51)	0.01 **
Neurological disease, n (%)	24 (8)	18 (10)	6 (5)	0.18 **
Trauma, n (%)	83 (30)	62 (37)	21 (19)	<0.01 **
Cardiovascular disease, n (%)	55 (20)	28 (17)	27 (24)	0.15 **
**Comorbidity **				
Hypertension, n (%)	54 (20)	27 (16)	27 (24)	0.12**
Diabetes mellitus, (%)	13 (4)	6 (4)	7 (6)	0.44**
Congestive heart failure, n (%)	15 (6)	9 (5)	6 (5)	0.79 **
Two or more comorbidities	25 (9)	11 (6)	14 (13)	0.13 **
**Type of infection **				
VAP, n (%)	200 (72)	118 (70)	82 (74)	0.6 **
CVC related-BSI, n (%)	62 (23)	39 (23)	23 (21)	0.73 **
Other, n (%)	17 (5)	11 (7)^c^	6 (5) ^d^	0.89 **
**Treatment **				
CMS, n (%)	90 (32)	59 (35)	31 (28)	0.25**
CMS+NAs, n (%)	57 (20)	31 (18)	26 (23)	0.39 **
CMS+ glycopeptides alone ^e^, n (%)	39 (68)	23 (59)	16 (41)	1***
CMS + aminoglicoside alone^e^, n (%)	8 (14)	5 (62.5)	3 (37.5)	
NAs, n (%)	132 (47)	78 (47)	54 (48)	0.8 **
**Outcome**				
Days in ICU ^b^	23 (13 to 37)	22 (13 to 34)	25 (14 to 42)	0.16 *
ICU mortality - n (%)	116 (41)	38 (23)	78 (70)	<0.01 **

*^a ^*Differences between subgroups with and without AKI onset after ICU admission: *Mann-Whitney test; **Chi-squared test, *** Fisher's exact test

*^b ^*Values are given as the median (interquartile range).

*^c ^*wound infection n = 2, abdominal abscess n = 5, urinary tract infection n = 2, meningitis n = 2

*^d ^*wound infection n = 1, abdominal abscess n = 3, urinary tract infection n = 2

*^e ^*in this analysis were excluded the patients who received aminoglycoside + glycopeptide ( n = 10) (18%)

One hundred thirty-two of the patients received intravenous therapy with NAs alone (glycopeptides and aminoglycosides). Eight (6%) of these patients received two nephrotoxic antimicrobials.

The other 147 were treated intravenously with CMS, alone (CMS group, n = 90) or with one or more NAs (CMS + NAs group, n = 57). The NAs in the latter group were vancomycin in 39 cases, vancomycin plus amikacin in 7, amikacin in 5, gentamicin in 3, and vancomycin plus gentamicin in 3. In all cases, the infection was associated with at least one bacterial isolate that displayed persistent *in vitro *susceptibility to colistin only. In the subgroup that was also receiving NAs, patients also had one or more isolates displaying susceptibility to the specific NA being administered.

CMS was administered as Colimicina^® ^(UCB Pharma SpA, Milan, Italy); 1 million UI per vial). All 147 patients received a loading dose of CMS (4 million IU) followed by a daily dose of 130,000 IU per kilogram of ideal body weight (IBW) (divided into three doses per day) [12]. For patients with creatinine clearance of less than 70 mL/min but more than 30 mL/min one-third of the normal daily dose twice a day (for example, 6 million IU divided into two doses per day for a 70 Kg patient); with a creatinine clearance <30 mL/min one-third of the normal daily dose once a day (for example, 3 million IU once a day for a 70 Kg patient); during CRRT we used one-third of the normal daily dose twice a day [13,14].

The median length of CMS therapy was 11 days; the cumulative CMS dose was 93.999.975 IU, and there were no significant differences between the CMS and CMS + NA subgroups involving any of these variables (*P *= 0.26 and *P *= 0.37, respectively)

Normogram based on creatinine clearance (CLCr) was used for calculation of the vancomycin daily dosage administered by continuous infusion in order to target a steady state concentration (CSS) at 15 to 20 mg/L [15]. We, therefore, confirm our dosage monitoring the serum vancomicin concentration. Aminoglycoside were administered according to one daily dosing schedule of 20 mg/kg/day for Amikacin and 5 mg/kg/day for Gentamycin; dosage was confirmed by monitoring the serum concentration and was adjusted as a function of CLCr. The target trough (1 µg/mL) was easily achieved using a once daily dose [16]

A total of 116 (41%) of the patients died while they were in ICU (Table 1).

One hundred eleven (40%) of the 279 patients developed AKI during their stay in the ICU. In the NAs group, 10% the AKI cases were classified as Risk, 13% as Injury and 18% as Failure; in the CMS group, 6% of the cases were classified as Risk, 7% as Injury and 22% as Failure; and in the CMS + NAs group, 7% of the AKI cases classified as Risk, 12% as Injury and 26% as Failure. The median onset of AKI was 10 days (8 to 15) (25^th ^to 75^th^) in the CMS group, 11 days (10 to 12) (25^th ^to 75^th^) in the NAs group, 12 days (10 to 21) (25^th ^to 75^th^) in the CMS + NAs group. Compared with the non-AKI subgroup, those who developed AKI were significantly older and had significantly higher SAPS II scores. In addition, septic shock rates and ICU mortality were roughly three times higher than those in the non-AKI group; in fact, in the AKI group, the ICU mortality and septic shock rates were 70% and 74%, respectively (Table 2). The vast majority of AKI patients had an albumin serum level less than 2 g/dL (Table 2). The vast majority of the infections considered in this study were ventilator-associated pneumonia (VAP) or catheter-related bloodstream infections (CRBSIs), and in almost half of all cases (46%), septic shock was present at infection onset.

We did not find any difference in the incidence of AKI in respect to the etiology of infections among the three groups studied. Nine out of 17 Failure patients who survived were discharged from ICU as Failure but without a dialysis prescription; 1 as Injury, 4 as Risk and 3 with a complete recovery of the renal function. Five out of 7 Injury patients who survived were discharged from ICU as Injury and 2 as Risk. Five out of 9 Risk patients who survived were discharged from ICU as Risk and 4 with a complete recovery of the renal function (Table 3).

**Table 3:** Outcome at the ICU discharge of AKI patients

AKI during ICU stay	Outcome at ICU discharge				
	**Normal **(n)	**Risk **(n)	**Injury **(n)	**Failure **(n)	**Dead **(n)

**Risk **n = 22	4	5	0	0	12
**Injury **n = 30	0	2	5	0	21
**Failure **n = 59	3	4	1	9	45

The results of the logistic regression are shown in Tables 3, 4 and 5. In the complete study population (n = 279), the multivariate analysis showed that SAPS II scores and the presence of septic shock at infection onset were independently associated with AKI. The other significant variables at the univariate analysis were not included in the multivariate final logistic model using a stepwise procedure. A ROC curve analysis was performed to assess the accuracy of the final regression model showing AUC ± SE = 0.79 ± 0.03 with 95% C.I. 0.74 to 0.84; Chi Square statistics: *P *<0.001 (Table 4). In the 147 patients who received CMS (Table 5), the likelihood of developing AKI was not significantly different in the CMS and CMS + NA subgroups. In contrast, onset of AKI was two times more likely in patients with a SAPS II score ≥43 and six times more likely in those whose infections had presented with septic shock. A ROC curve analysis was performed to assess the accuracy of the final regression model showing AUC ± SE = 0.76 ± 0.04 with 95% CI 0.7 to 0.8; Chi-square statistics *P *<0.001.

**Table 4:** Logistic regression analysis of factor associated with AKI in the study cohort

	Univariate analysis			Multivariate analysis		
**Variables**	**O.R**.	**95% C.I**.	***P*-value**	**O.R**.	**95% C.I**.	***P*-value**

Age, years	1.02	1.01 to 1.04	<0.01			
SAPS II score	1.04	1.03 to 1.06	<0.01	1.03	1.01 to 1.05	<0.01
Female	0.63	0.37 to 1.06	0.08	0.62	0.34 to 1.14	0.12
Septic shock at infection onset	7.5	4.36 to 12.9	<0.01	5.89	3.35 to 10.35	<0.01
Albumin serum levels <2 g/dL	2.66	1.32 to 5.37	<0.01			
Total bilirubin serum levels >5 mg/dL	2.2	0.85 to 5.65	0.1			
NSAID	0.62	0.35 to 1.1	0.1			
ACEI	1.83	0.89 to 3.78	0.1			
i.v. iodate contrast	1.27	0.76 to 2.1	0.35			
Immunocompromised status	1.14	0.63 to 2.05	0.64			
**Causes of ICU admission**						
Sepsis	1.9	1.17 to 3.1	<0.01	1.74	0.99 - 3.05	0.052
Neurological disease	0.47	0.18 to 1.24	0.13			
Trauma	0.4	0.22 to 0.7	<0.01			
Cardiovascular disease	1.61	0.89 to 2.9	0.12			
**Co-morbidities**						
Hypertension	1.68	0.92 to 3.05	0.09			
Diabetes mellitus	1.79	0.58 to 5.47	0.31			
Congestive heart failure	1	0.35 to 2.92	0.99			
Two or more comorbidities	2.06	0.9 to 4.72	0.08			
**Type of infection**						
VAP	1.19	0.7 to 2.05	0.51			
CVC related-BSI	0.86	0.48 to 1.55	0.62			
Other	0.81	0.29 to 2.27	0.7			
Treatment with CMS (vs. NAs)	0.91	0.56 to 1.47	0.7			

The variables included in the final predictive model were selected with a stepwise procedure: albumin serum levels <2 g/dL, total bilirubin serum levels >5 mg/dL, ACE inhibitors, NSAID, age, neurological disease, trauma, cardiovascular disease, hypertension and two or more co-morbidities were variables not included in the final model. We assessed discrimination of the model with area under the receiver operating curve (AUC ± SE = 0.79 ± 0.03 with 95% C.I. 0.74 to 0.84; Chi Square statistics: *P *<0.001).

ACEI, angiotensin converter enzyme inhibitor; AUC, area under the curve; BSI, bloodstream infection; CI, confidence interval; CMS, colistin methanesulfate; CVC, central venous catheter; NAs, other nephrotoxic antibiotics (aminoglycosides, glycopeptides); NSAID, nonsteroidal anti-inflammatory drug; SAPS II, Simplified Acute Physiology Score Two (calculated 24 h after ICU admission); VAP, ventilator-associated pneumonia.

**Table 5:** Logistic regression analysis of factors associated with AKI in patients who received CMS and CMS/NAs.

		Univariate analysis			Multivariate analysis^b^		
**Variables**	**No. AKI/total (%)**	**O.R**.	**95% C.I**.	***P*-value**	**O.R**.	**95% C.I**.	***P*-value**

**Age, years ^a ^**							
<55	23/71 (32)	1.00					
≥55	34/76 (45)	1.68	0.86-3.31	0.13			
**Sex**							
Male	39/95 (41)	1.00					
Female	18/52 (34)	0.76	0.37-1.53	0.44			
**SAPS II ^a ^**							
<43	20/73 (27)	1.00					
≥43	37/74 (50)	2.65	1.33-5.27	<0.01	2.26	1.07-4.79	0.03
**Septic shock at Infection onset **							
No	20/89 (22)	1.00					
Yes	37/58 (64)	6.1	2.92-12.62	<0.01	5.64	2.66-11.94	<0.01
**Treatment with CMS**							
CMS with NAs	26/57 (45)	1.00					
CMS alone	31/90 (34)	0.62	0.31-1.23	0.17			
**Cumulative CMS dose ^a,c^**							
<93.999.975 (IU)	33/73 (45)	1.00					
≥93.999.975 (IU)	24/74 (32)	0.58	0.29-1.13	0.11	0.61	0.29-1.29	0.19
**Duration of CMS therapy^a ^**							
<11 days	30/72 (42)	1.00					
≥11 days	27/35 (36)	0.78	0.4-1.53	0.48			

The variables included in the final predictive model were selected with a stepwise procedure: age and treatment with CMS were not included in the final model.

***^a ^***age, SAPS II, duration of CMS therapy, and cumulative CMS dose were dichotomized around median values. ***^b ^***The ROC curve analysis was used to assess the goodness of the final logistic regression model (AUC ± SE = 0.76 ± 0.04 with 95% CI 0.7 to 0.8; Chi-square statistics *P *<0.001). *^c ^*Includes loading dose of 4,000,000 IU.

AKI, acute kidney injury; AUC, area under the curve; CI, confidence interval; CMS, colistin methanesulfate; IBW, ideal body weight; IU, international unit; NAs, nephrotoxic antibiotics (aminoglycosides, glycopeptides); ROC, receiver operating characteristic; SAPS II, Simplified Acute Physiology Score II (calculated 24 h after ICU admission).

A similar picture emerged when we analyzed the 222 patients who received CMS alone (n = 90) or NAs alone (n = 132) (Table 6). The only independent predictors of AKI in this group were SAPS II scores ≥44 and septic shock at infection onset. A ROC curve analysis was performed to assess the accuracy of the final regression model showing AUC = 0.8 ± 0.03 with 0.75 to 0.86 95% CI; Chi-square statistics: *P *<0.01.

**Table 6:** Logistic regression analysis of factors associated with AKI in patients who received CMS and NAs

		Univariate analysis			Multivariate analysis^b^		
**Variables**	**No. AKI/total (%)**	**O.R**.	**95% C.I**.	***P*-value**	**O.R**.	**95% C.I**.	***P*-value**

**Age, years ^a ^**							
<64	36/110 (32)	1.00					
≥64	49/112 (43)	1.59	0.92-2.75	0.09			
**Sex**							
Male	63/150 (42)	1.00					
Female	22/72 (30)	0.6	0.33-1.1	0.1	0.6	0.29-1.2	0.15
**SAPS II ^a ^**							
<44	25/109 (22)	1.00					
≥44	60/113 (53)	3.8	2.13-6.79	<0.01	2.45	1.17-4.74	<0.01
**Septic shock at Infection onset **							
No	19/121 (15)	1.00					
Yes	66/101 (65)	10.12	5.34-19.12	<0.01	8.24	4.26-15.93	<0.01
**Treatment with CMS**							
No	54/132 (41)	1.00					
Yes	31/90 (34)	0.75	0.43-1.32	0.33			

The variables included in the final predictive model were selected with a stepwise procedure: age was not included in the final model.

*^a ^*Age, SAPS II were dichotomized around median values.

***^b^***The ROC curve analysis was used to assess the goodness of the final logistic regression model (AUC = 0.8 ± 0.03 with 0.75 to 0.86 95% CI; Chi-square statistics: *P *<0.01)

AKI, acute kidney injury; AUC, area under the curve; CI, confidence interval; CMS, colistin methanesulfate; NAs, nephrotoxic antibiotics (aminoglycosides, glycopeptides); ROC, receiver operating characteristic; SAPS II, Simplified Acute Physiology Score Two (calculated 24 h after ICU admission).

These findings indicate that in ICU patients without pre-existing renal disease who require nephrotoxic antimicrobial drug therapy for XDR bacterial infections, the use of CMS - with or without NAs - does not significantly increase the risk for AKI over that associated with NAs therapy alone.

## Discussion

The cohort treated with high doses of CMS for nosocomial XDR infections in our study represented approximately 10% of the entire population admitted to the general ICUs during the two-year study period. The overall incidence of AKI in the 279 cases we analyzed was 40%, and there were no significant differences among rates observed in the CMS (34%), CMS+NAs (45%) and NAs subgroups (41%). These data are consistent with the results of the Nefroint study [8], a multicenter study conducted in Italian ICUs: in the subgroup of 133 patients without AKI at ICU admission, the incidence of AKI was 40% regardless of whether or not nephrotoxic drugs were administered. A recent meta-analysis [17] on six controlled studies comparing colistin vs other antibiotics for treatment of VAP in patients without cystic fibrosis suggested that colistin may be as safe as other standard antibiotics used for these drug-resistant infections. In particular, the nephrotoxicity rate for colistin was similar to that in the control group.

Our multivariate analysis revealed that SAPS II scores ≥43 and the presence of septic shock at infection onset were independently associated with AKI, but high-dose intravenous CMS therapy for more than seven days was not a risk factor for development of new onset AKI.

Since CMS's recent re-emergence as a last-resort treatment of infections caused by XDR pathogens (including *Acinetobacter baumanii, Pseudomonas aeruginosa *and *Klebsiella pneumonia*), many authors have investigated its adverse effects, in particular, its potential nephrotoxicity. Like aminoglycosides, the polymyxins cause damage to the kidneys at the level of the proximal tubules, where both classes of drugs are extensively reabsorbed via the endocytic receptor protein megalin [18]. Colistin was originally used in the 1960s to combat infections caused by gram-negative bacteria, but it was abandoned in the 1970s because of its reported association with high nephrotoxicity rates [13,19] and because new, apparently less toxic antibiotics (for example, the aminoglycosides) were becoming available. Several studies published over the past decade, however, have demonstrated that CMS is not associated with serious adverse effects, and although nephrotoxicity incidence rates varied (0% to 32%) [20-24], they were clearly lower than those reported in the 1960s and 1970s. The differences between older and more recent findings have been attributed to various factors, including the increased presence of chemical impurities in older colistin preparations, the variable definitions of acute renal impairment used in the various studies, closer monitoring and, last but not least, the improved maintenance of patient hydration by today's physicians [20].

Research endorsed by the Acute Dialysis Quality Initiative led to the publication of the RIFLE classification, with standardized criteria for various degrees of renal dysfunction [6]. The RIFLE approach can detect AKI with high sensitivity and high specificity. It can also be used to predict the prognosis of affected patients, and it provides a useful framework for comparing the results of different studies. Two recent studies that used the RIFLE criteria to investigate colistin-related nephrotoxicity [25,26] documented high incidences of mild renal impairment (about 43%) in both cohorts (even though the two populations differed in terms of illness severity).

Indeed, in most recent studies, the colistin-treated populations have been heterogeneous in terms of baseline illness severity, baseline renal function and treatment variables, including daily doses and duration of treatment. Daily doses of CMS used in these studies ranged from 3 million to 11 million IU [21,25,27-30]. To make matters worse, there is also wide variation involving the type of preparation used, that is, CMS (where 2 million IUs correspond to 160 mg of the drug) versus colistin base (where 2 million IUs equals 60 mg). The importance of universal dosing terminology has been emphasized by several investigators [19,20].

This antibiotic is being used with increasing frequency to treat critically ill patients - despite the absence of clinical guidelines and dosing recommendations for this particular population. Multi-organ dysfunction and severe XDR infections can alter the pharmacokinetics of CMS and colistin in terms of half-life and rates of formation of colistin from CMS [30], and the larger volumes of distribution present in these critically ill septic patients can cause a lower concentration of the antibiotic [5]. Early and appropriate goal-directed fluid therapy is fundamental in acute resuscitation of these critically ill patients; however, it is almost always associated with a certain degree of fluid overload, especially in septic patients, which promotes tissue edema that could potentially contribute, itself, to progressive organ dysfunction. Both fluid balance and urine volume are independent predictors of mortality in adult critically ill patients with AKI [31].

Plachouras *et al*. [12] studied the pharmacokinetics of intravenously administered CMS in critically ill patients and concluded that a loading dose of at least 9 million IU of CMS is needed in these cases to produce plasma concentrations of the drug within the minimum inhibitory concentration (MIC) range indicative of susceptibility. Failure to achieve such concentrations can lead to the emergence of resistant strains, and it can also result in increased mortality.

In light of these findings, we decided to investigate the nephrotoxicity of high-dose CMS therapy, in terms of RIFLE-defined AKI, in patients with no AKI at baseline. Hartzell and collaborators [25] used a similar approach in a young and otherwise healthy population of patients on a general medicine ward. The patients had no other confounding comorbidity, but the mean duration of CMS therapy was longer than it was in our study. The authors found a significant association between the cumulative CMS dose and the risk of nephrotoxicity in patients receiving CMS for more than 14 days. This finding contrasts with the results of our logistic regression analysis, which showed that neither the cumulative CMS dose nor the duration of treatment was a risk factor for developing new-onset AKI in severely ill ICU patients. The median days of CMS treatment of our patients, however, was lower that than reported by Hartzell and collaborators and could probably justify the discordance with our results as well as the difference in severity of the two population studied. We agree, nevertheless, that creatinine levels need to be closely monitored in patients receiving prolonged treatment with CMS.

Pogue *et al*. [26] reported that CMS nephrotoxicity is related to the daily dose but not to cumulative exposure. However, the population they studied was heterogeneous in terms of pre-treatment renal function. Furthermore, although illness severity scores were not reported, their patients were probably not as critically ill as ours. Only 14% had septic shock, 15% were on vasopressors and only 62% were being mechanically ventilated. These are important differences because, as noted above, the pharmacokinetics of CMS and colistin are different in the critically ill [5,29], and in our study a SAPS II score ≥43 was independently associated with AKI. Comparison of findings in ICU and non-ICU cohorts is also complicated by the fact that the former patients are likely to be more closely monitored and more rapidly treated than those being cared for on general wards.

Septic shock was the strongest predictor of AKI in our cohort. Many authors agree that the renal effects of sepsis, *per se*, should not be underestimated. Early AKI is common in septic shock [32], and it may potentiate the effects of colistin and other drugs on the kidney. The combination of septic shock and AKI had a consistently negative effect on survival. Among the patients with septic shock who did not receive CMS at all (that is, those who were treated with NAs alone), the incidence of AKI was 64% (45/70), which confirms the predominant role of septic shock in the kidney injury (Figure 1).

### Limitation of the study

The main limitation of our study is its retrospective design. This shortcoming is partially compensated for by the large number of patients studied, but it is almost impossible to avoid. A prospective randomized trial would inevitably be associated with ethical problems since for many infections, colistin is the only treatment option.

None of the patients received the CMS as empirical therapy and a definitive appropriate therapy was achieved within 36/48 hours. There are no data showing that using CMS as an empiric regimen could reduce the risk of inappropriate therapy and/or could reduce the incidence of septic shock or increase the risk of AKI. These interesting issues should be tested by specific trials.

## Conclusions

In conclusion, in ICU patients with normal baseline renal function who receive CMS and/or NAs, the incidence of AKI as defined by the RIFLE classification is clearly high - 40%. However, high-dose CMS therapy does not appear to be a risk factor for this outcome. Instead, the development of AKI was strongly correlated with the presence of septic shock and with the severity of the patients' underlying illness, as reflected by the SAPS II score. These findings suggest that renal protection measures, such as blood volume maintenance, are of utmost importance in critically ill patients with infections that require treatment with CMS.

## Key messages

• The incidence of AKI in critically ill patients without pre-existing renal diseases is strongly correlated with the presence of septic shock and with illness severity.

• Compared with other nephrotoxic antimicrobials, high-dose CMS does not appear to increase the risk of new-onset AKI in this setting.

## Abbreviations

ACEI: angiotensin converter enzyme inhibitor; ADQI: Acute Dialysis Quality Initiative; AKI: acute kidney injury; BSI: bloodstream infection; CMS: colistin methanesulfonate; ClCr: creatinine clearance; CRBSIs: catheter-related bloodstream infections; CRRT: continuous renal replacement therapy; CSS: steady state concentration; IBW: ideal body weight; ICU: intensive care unit; IQR: interquartile ranges; IU: international units; i.v.: intravenous; GFR: glomerular filtration rate; MDRD: Modification of Diet in the Renal Disease; MIC: minimum inhibitory concentration; NAs: nephrotoxic antimicrobials; NSAID: nonsteroidal anti-inflammation drug; ORs: odds ratios; RIFLE: Risk Injury-Failure-Loss-End-stage kidney disease; ROC: receiver operating characteristic; SAPS II: Simplified Acute Physiology Score II; Scr: serum creatinine; VAP: ventilator associated pneumonia; XDR: extensively drug-resistant

## Competing interests

The authors have no competing interests to declare relative to this article.

## Authors' contributions

MR, LM, EA, MV, PP and MA designed the study. AL, MV, GR and GDP collected and assembled the data. LM and EA performed the statistical analysis. MR, LM, MA, EA, PP and AL drafted the manuscript, and all authors have read and approved the final manuscript.
